# Maternal and child access to care and intensity of conflict in the occupied Palestinian territory: a pseudo longitudinal analysis (2000–2014)

**DOI:** 10.1186/s13031-019-0220-2

**Published:** 2019-08-07

**Authors:** Tiziana Leone, Diego Alburez-Gutierrez, Rula Ghandour, Ernestina Coast, Rita Giacaman

**Affiliations:** 1 Department of International Development, London School of Economics and Political Science, London, UK; 2 Department of Social Policy, London School of Economics and Political Science, London, UK; 3 Institute of Community and Public Health University of Birzeit, Birzeit, State of Palestine; 4 Present Address: Max Plank Institute for Demographic Research, Rostock, Germany

**Keywords:** Conflict, Palestine, Vaccination, Maternal health, Barriers

## Abstract

**Background:**

In the occupied Palestinian territory (oPt), access to maternal and child healthcare (MCH) services are constrained due to the prolonged Israeli military occupation, the Separation Wall, army checkpoints, and restrictions on the movement of people and goods. This study assesses the relationship between conflict intensity and access to Maternal and Child Health care in occupied Palestinian territory (oPt). To the best of our knowledge, the impact of conflict on access to health care has not been measured due to the lack of data.

**Methods:**

We analyse pooled data from household surveys covering a fifteen-year period (2000–2014) of children (*n* = 16,793) and women (*n* = 8477) in five regions of the oPt. Conflict intensity was used as a continuous variable defined as the square root of non-combatant conflict mortality taken from monthly death rates of non-combatants by region. We use multilevel logistic models to explain four outputs: child vaccination schedules, antenatal care, caesarean sections, and complications during pregnancy.

**Results:**

Locality is important with results showing the negative impact of conflict intensity on access to care, especially in the South West Bank for maternal health services and Central West Bank for vaccination (B − 0.161 *p* = 0.000 for DPT). Wealth is only significant for DPT vaccinations with poorest (B − 0.098 *p* = 0.005) and poor (B − 0.148 *p* = 0.002) individuals less likely to access services. Otherwise conflict does not show a differential effect across socio-economic conditions.

**Conclusions:**

This study shows how locality is the strongest factor when looking at the impact of conflict in the oPt. Preventative services (ANC and vaccinations) are the most affected by conflict. We recommend a greater use of community health care to improve access to maternal and child care when barriers impede access to health facilities during times of conflict.

## Background

The direct consequences of conflict on health are usually established by numbers and rates of casualties and long term disabilities. Much less attention is paid to the indirect impact^1^ of conflict on health of non-combatant populations [1]. We know that conflict undermines a country’s health care capacity and disrupts the delivery of basic health services. Yet, the consequences of conflict on access to healthcare are difficult to quantify, mainly due to a lack of adequate data [2].

In the occupied Palestinian territory (oPt) the continued Israeli military occupation, the Separation Wall, Israeli army checkpoints, and the restrictions of the movement of people and goods have limited the access of Palestinians to healthcare services [3]. This conflict is unique due to its protracted nature spanning decades, with variations in the intensity and nature of the conflict [3–5]. The instability of the territory has been further marred by a series of political events which have been linked to periods of increased tension and conflict (Table 1), such as changing UN resolutions or elections in Israel.

**Table 1:** Palestine conflict and political timeline 1967–2014

1967	The West Bank, including East Jerusalem and the Gaza Strip come under Israeli military occupation
1987	The first Palestinian Intifada (uprising) begins, and lasts till 1993
1993	Oslo Accords, establishment of Interim Palestinian Authority in charge of administration and health care
1994	Palestinian Ministry of Health established
2000	Interim political solution collapses. Beginning of second Palestinian Intifada (uprising)
2002	Israeli incursions into the West Bank and ransacking of several educational, cultural, and administrative institutions including the Palestinian Central Bureau of Statistics
2004	Gaza Strip-Egypt border constructed. Egypt controls south of the Strip
2005	Israel dismantles settlements in the Gaza Strip but control over external perimeter of the Strip and siege conditions remain
2006–08	Democratic election of Hamas to majority in the Palestinian National Authority. Boycott of the elected government by several foreign countries, Israel withholds tax revenues which form 75% of oPt revenues. Two Israeli military operations in the Gaza Strip
2007	Hamas takes over the Gaza Strip
Nov 08-Jan 09	Israel Invasion of the Gaza Strip
2012	Israeli military operations in oPt
2014	Israel-Gaza conflict
2014	Israeli invasion of the Gaza Strip
2014	Compromise Hamas Fatah government unity. Israel-Gaza conflict

One of the key features of the Palestinian-Israel conflict in relation to access to healthcare, lies in geographic divisions and checkpoints that can be either permanent or temporarily erected at the time of heightened conflict. After the 1993 Oslo Accords (see Table 1), Palestine was divided into territory A, B, and C. The Palestinian National Authority (PNA) was responsible for zone A (main urban areas in the West Bank), whilst Zone B was shared between PNA and Israel. Zone C which represents the remaining 60% of the territory is completely controlled by Israel. This includes most Israeli settlements and agricultural land [3].^2^ Access to East Jerusalem (area C), where the major Palestinian hospitals are located, is largely restricted to local residents with an Israeli-issued Jerusalem identity card [6]. This fragmentation of the territory makes movement within the West Bank challenging even in the absence of violent conflict.

The impact of Israeli occupation of the oPt on maternal and child health care has been explored at the macro level for the whole territory, both the West Bank and Gaza Strip [7]. The authors show how living conditions in oPt have deteriorated and how conflict, increasing poverty and unemployment have affected access to MCH. However, no research thus far has not analysed data at the micro level for women and children accounting for socio-economic background, nor has it considered the longitudinal impact of conflict. Other studies of health care access in oPt rely on ad hoc data collected sub-nationally which are not representative of the overall population, and frequently exclude the Gaza Strip. This is due to the challenges of collecting data for the country as a whole, and in particular in the Gaza Strip. Studies of healthcare access in oPt often rely on data collected for other purposes, using compiled information on road blockages [8] or Geographical Information Systems (GIS) [9]. These data often become out of date very quickly due to the frequent changes imposed by the Israeli military on the movement of people and goods. The frequency of the data on roadblocks is patchy and not consistent across time. Thus, data on blockages or GIS tend to be of lower quality than data on casualties and tend to be less complete.

The aim of this study is to establish the relationship between intensity of conflict and maternal and child health care access in the oPt between the Second Palestinian Uprising (Intifada) of 2000–2004 and 2014. We hypothesise that the changing conflict intensity has had effects on maternal and child healthcare access in the oPt. In particular, we focus on antenatal services, pregnancy complications, intrapartum services, and post-partum using vaccinations as a proxy for child healthcare.

Access to maternal and child health care (MCH) is a core element of a functioning health system and is often used as a key indicator for evaluation [10]. The state of maternal and child health is an important indicator defining the overall developmental state of a country, recognised by the inclusion of targets related to MCH in the Millennium Development Goals (4 and 5) and Sustainable Development Goals [3, 11].

Above all, adequate access to maternal and child healthcare requires an uninterrupted supply of services that are affordable, reachable, and contextually and culturally acceptable [12]. Within a conflict setting access is affected in multiple ways: supplies are interrupted or reduced; specialised staff is difficult to train and retain; and, physical access is reduced due to damaged infrastructure and barriers. In addition, psychological barriers to access are increased as fears for safety can prevent people from trying to access services or alter the frequency/timing of use [13].

Exposure to armed conflict has repercussions on the civilian population and in particular on vulnerable populations such as pregnant women and children. It is widely reported that prenatal stress due to conflict has an impact on foetal development, pregnancy complications and pregnancy outcomes [14]. The situation is exacerbated by limited access to healthcare including antenatal care, hospital deliveries, and access to key services such as caesarean sections and assisted delivery (.e.: forceps and ventouse) [15]. Conflicts have been found to exacerbate and reignite preventable diseases with vaccinations due to shortage of supplies and to physical barriers to access [16, 17]. This paper focuses on the impact of conflict on the demand side for services, with a particular focus on MCH.

### Context

MCH provision in the oPt has seen a shifting in governance and supply since the 1960s. Provision was increasingly institutionalised in the oPt after 1967, when health policy was directed by the Israeli Civil Administration. Hospital childbirth became more prevalent after 1980, under the premise that deliveries attended by traditional birth attendants (*dayat*) resulted in higher infant mortality [18].

Since the Oslo Accordsin 1993 and the establishment of the Palestinian Authority in 1994, healthcare in the West Bank has been administered by the Ministry of Health and services are delivered in collaboration with UN Relief and Works Agency for Palestine (UNRWA), Non-Governmental Organisations (NGOs), the military medical services, and the Palestinian Red Crescent [8, 19]. The situation being very different in the Gaza Strip and the West Bank. The West Bank has been able to implement a programme of reforms in health whereas healthcare in Gaza Strip has been on the brink of collapse mainly due to the siege conditions imposed by Israel and Egypt [5].

Antenatal care and immunisation coverage in the oPt are similar to other countries in the region. In 2005, over 95% of women had at least one ANC visit and vaccination coverage for children aged below 5 is similarly high [8]. Nearly all (99.6%) all deliveries are conducted by a skilled birth attendant (World Bank, 2017). Immunizations and births in a government hospital have been free of charge since 2000 [7]; 53.6% of all births in 2016 were in a government hospital. Unpublished estimates suggest that 82% of women were covered by the national insurance in 2011 [20]. However, although maternal and infant mortality have declined significantly in the oPt since the 1960s, rates are still high relative to Israel and Lebanon (Table 2). Maternal mortality showed a slight increase in 2014, but this could be due to measurement errors as a result of particularly challenging data collection circumstances [19]. The oPt performs worse than Israel on all MCH indicators and comes second to Jordan only for the Maternal Mortality Ratio (MMR).

**Table 2:** Demographic and socio-economic background oPt: and selected countries, 2017

	oPt			Israel	Jordan	Lebanon
	West Bank (WB)	Gaza Strip (GS)	Combined WB + GS			
Population in millions	2.8	1.8	4.6	8.5	9.4	6.0
Total Fertility Rate	3.7	4.5	4.1	3.1	3.4	1.7
Maternal Mortality Ratio (per 100,000)	19.8	30.6	24.7	5.0	58.0	15.0
Mortality Under 5 (per 1000)	17	22	20	5	18	8
GDP per capita (US$)	2015	1103	1766	37,901	2902	11,930
Unemployment rate	18.0%	38.0%	24.9%	5.6%	11.9%	6.3%

Source: World Bank 2017

Health financing is patchy within the oPt. The main scheme of health coverage is a government health insurance scheme that individuals have to pay in to. Only civilians and retirees are mandated to pay in, and itt is estimated around 30% of individuals who should contribute are not, adding to the strain on the system [8]. In order to give birth free of charge in a public hospital women need to have government health insurance coverage in the West Bank.

The stalling or reverse of progress has been particularly significant since the Second Intifada in 2002–04, especially during periods of increased conflict [5]. Access to healthcare might be affected by increased restrictions on movement and international aid.

The building of the Separation Wall, which began in 2002 (440 km built by 2014), has made movements across the oPt and between the oPt and Israel more difficult. Additional Israeli army checkpoints and barriers have added to the difficulties of movement within the oPt. Home demolitions and confiscation of lands have increased the Palestinian population’s psychological stressors, including not knowing when crossing will be allowed and whether healthcare can be sought if in need [6, 21].^3^

The oPt faces economic constraints which have impacted healthcare spending, including for medical products and payroll of healthcare workers. The Ministry of Health, has been described as “insufficient and unsystematic” given the complex interactions between internal forces—as political power consolidated within the Palestinian National Authority—and external factors related to the protracted conflict and negotiations with the agendas of international donors [18]. In the Gaza Strip, the quality of health provision decreased substantially after the election of the Islamic Resistance Movement (Hamas) in 2006 and the subsequent boycott of this government by international donors, in addition to the siege imposed by Israel. Gaza has additional supply side burdens, with several hospitals seriously damaged or destroyed by bombing (eg.: 2008–2009 and 2014^4^), stalled training of health professionals, and limits set by Israel on the quantity and type of health supplies permitted to go through checkpoints [22, 23]. Fewer than 200 people and less than one truckload of goods per day were allowed out of Gaza via Israel during the first half of 2013 [6]. Gaza patients need to apply for permission to travel between 7 and 10 days prior to a hospital appointment and if their travel is approved, they are informed the night before their appointment [6]. All this adds to a health system in the Gaza Strip which is under acute strain and facing collapse. Locality is therefore key to understanding access to services in Palestine.

An extra layer in the Palestinian setting is that of refugees, their care is provided in large part by UNRWA [24], including one hospital in northern West Bank which is free of charge and open to the whole population. Refugee camps are often the first to be closed off during heightened conflict and camp residents have additional challenges when trying to access services [25, 26]. However, the situation of refugees in Palestine is more complex than other contexts. Refugees are often settled within communities with access to a variety of services which allows them at times to have a greater access to services than in other refugee camps found in other middle-Eastern countries [5].

Information on the health workforce involved in childbirth is scarce; however, the available information shows a low number of licenced midwives and *dayat* provide delivery services, especially at the community level [27, 28]. The midwife code of practice is considerably restricted in the oPt and there has been a general failure to employ community health workers to provide home based services such as maternal and neonatal care [7]. Most community midwives work in urban rather than rural areas and their presence is concentrated in Jenin and Hebron [27]. Midwives work with heavy caseloads, associated with the use of either C-section or oxytocin to speed labour in addition to increased use of medical interventions due to the extra stress of having to cross checkpoints during labour. Overall the shortage and lack of training for midwives further exacerbates the issues related with childbearing in the oPt [27].

For all Palestinians, psychological barriers to healthcare seeking derive from people’s unwillingness to have to go through Israeli army checkpoints and face delays, humiliation or fear of retaliation at each barrier crossing [4]. Fear of not making it to hospital as well as experiencing complications while at checkpoints add to the uncertainty of labour for Palestinian women [27]. A UN report during a period of enhanced conflict, reported that there had been an increase (8%) in home births, and that approximately 2500 women in labour a year were reporting difficulties crossing checkpoints [29]. However, there are no systematic data on births occurring at checkpoints as a result of delays and information remains anecdotal. It is plausible that non-emergency healthcare-seeking such as ANC and immunizations are also likely to be affected by such psychological barriers.

The rate of caesarean sections (C-sections) in the oPt has risen steadily in the last decade from 8.8% in 2000 to 20% in 2014. Abdul Rahim et al. (2009) suggest that this might reflect anxiety about healthcare access leading to women and practitioners wanting to control the timing of birth. To date, no analyses have tested the relationship between conflict intensity and the rate of C-sections empirically.

Overall the oPt is a setting where the relationships between health and conflict might differ from the major literature [5, 14–16]. First of all, given the size of the territory, distance is not an issue. The territory has plenty of health facilities but the country struggles with a shortage of health workforce in many areas outside Palestinian East Jerusalem and Ramallah. The protracted conflict is also characterised by road blockages and restriction to travel. It is therefore not feasible to analyse a confined period of time such as the studies done in Iraq or Afghanistan [14, 17]. Evidence also suggests that restrictions on movement have affected the oPt population access to health care irrespective of socio-economic status, although no statistical inference data is available to cross-check this descriptive result [18]. This is in contrast with previous studies in other countries which showed a higher impact on health and access to health for poorer strata of the population [1, 15].

## Methodology

### Data

Data for this study come from four surveys carried out by the Palestinian Central Bureau of Statistics (PCBS) between 2004 and 2014. The surveys were selected based on three criteria: data reliability (determined from personal communications with experts, review of the literature, and analysis of missing data), comparable sampling strategies, and the presence of comparable questions on maternal and child health. Six nationally-representative surveys with health data in Palestine were originally identified; the 1996 Palestinian Health Survey and the 2000 Demographic and Health Survey were excluded because they did not meet our inclusion criteria. The selected surveys include retrospective information about births. For maternal health outcomes, the questions refer to births in the two years before the survey; for immunisations the questions referred to three years before the survey. The resulting gaps in the continuous data (eg. 2000, 2007 and 2011 for maternal data) were overcome by pooling the surveys so that the comparison becomes more meaningful and the statistical power higher due to the larger sample size. This type of approach has been used to create pseudo-longitudinal data from different surveys [17, 30] but, to the best of our knowledge, it has never been applied to Palestinian data before.

Table 3 presents the four data sources used in our analyses. Data quality varies across surveys, but was assessed to be acceptable for the analyses through triangularisation and data checks (e.g. distributions by age, missing cases, outliers). Although slightly different sampling strategies were used in some of the surveys (see Table 3), personal communication with technical personnel at the PCBS confirmed that they were sufficiently comparable for our analyses. All surveys rely on enumeration areas derived from census data and apply weights on the observations in a similar way [31–34].

**Table 3:** Survey data sources included in analyses

Data	Demographic and Health Survey (DHS)	Palestinian Family Health Survey (PFHS)	Palestinian Family Survey (PFS)	Palestinian Multiple Indicator Cluster Survey (MICS)
Year of data collection	2004	2006	2010	2014
Sample size	6574	13,238	15,355	11,125
Sampling	stratified multi-stage	stratified two-stage random sample	multi-stage stratified cluster sample	stratified two-stage random sample
Non-response % individual level WB/GS	15.9/3.1	14.5/6.9	8.5/0.7	5.7^a^

^a^Non-response rates not reported separately for the West Bank and the Gaza Strip

Our analyses used four health outcome indicators, selected because they are harmonised across all surveys and because they represent key indicators of MCH access as well as one key indicator for maternal health status: child vaccination schedules (diphtheria, pertussis, and tetanus (DPT) and Oral Polio Vaccine (OPV)), number of antenatal visits (ANC) per pregnancy, C-sections, and complications during pregnancy.

Data availability restricted variable selection, exacerbated by inconsistent question wording across surveys and a lack of clarity on variable coding between datasets. Geographic analysis was limited to the regional level as governorate-level data are not available across all surveys. We decided to analyse at a higher geographical level (regions rather than governorates) in order to preserve the temporal continuity of the data which was important to get an account of the effect of conflict over time. Data for East Jerusalem were removed from all surveys as they were inconsistent across the datasets and because many holders of East Jerusalem IDs are able to access Israeli health centres, and therefore should not be compared with the rest of the oPt population. Given the small size of the regions considered, the level of detail of the analysis is still of significant in explaining variations in access and to exposure to conflict.

**Fig. 1:**
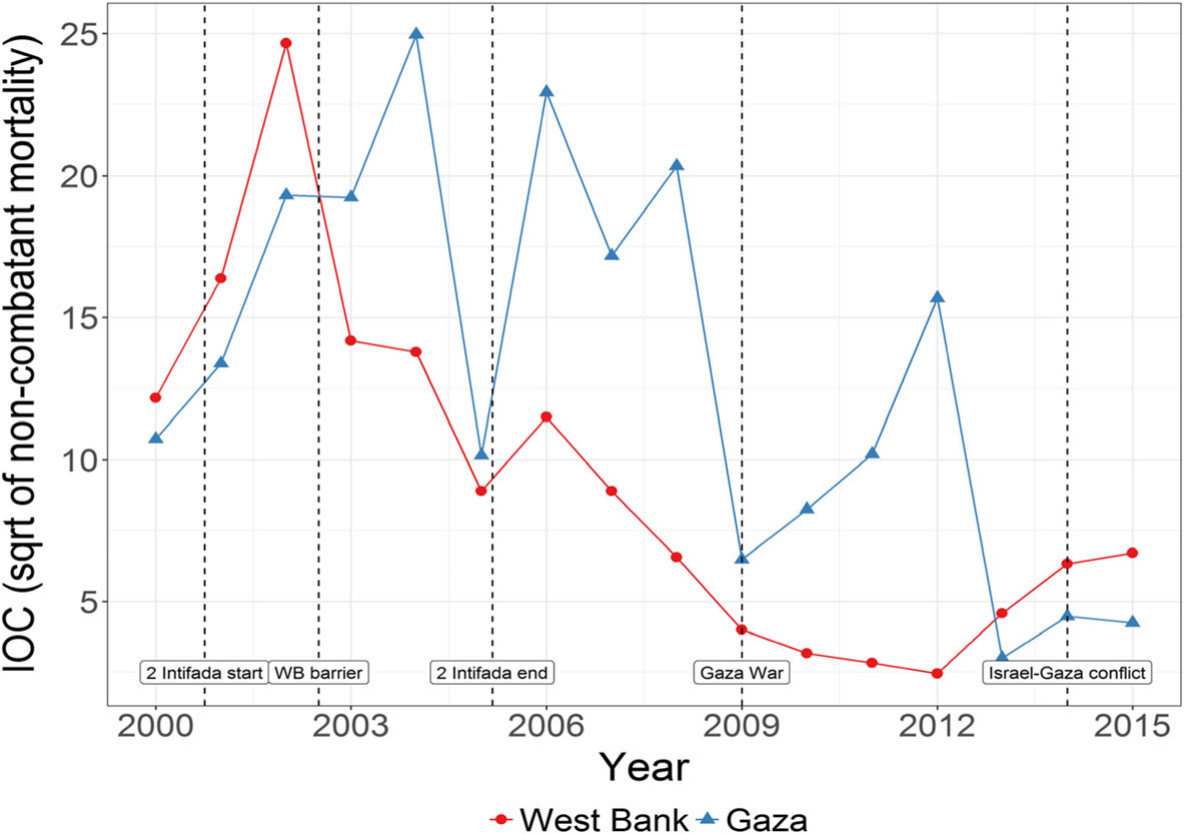
Intensity of conflict (IOC) by region in the oPt (2000–2014)

The variable *intensity of conflict* was defined as the square root of non-combatant conflict mortality in the five regions of the oPt (Fig. 1) using mortality data obtained from BT’selem (2016), considered to be the most objective and reliable data on conflict by both the Israelis and Palestinians [6]. Applying the principles of International Humanitarian Law during the early years of the Second Intifada, B’Tselem based its definition of casualties as non-combatants dependent on whether a person had taken actions against the Israeli occupying forces when killed or not [35]. The numbers include all individuals regardless of age. The variable is continuous, and we used the square root transformation due to lack of observations for some years in some regions in order to reduce skewness and to approximate the distribution of the variable to a Gaussian distribution. Previous studies have shown how square root transformations are commonly applied to count variables with small values that include zero [36]. This was appropriate for non-combatant casualties, since values tended to be small (see Table 7 in Appendix) and there were no casualties reported for certain region-month combinations in Palestine (e.g. no casualties were reported in the North West Bank between March 2009 and February 2010). Non-combatant conflict mortality is also a proxy for the level of conflict in the area generally defined by restrictions of movement and checkpoints. Separate sensitivity analyses (not shown) showed that restriction of movement (the number of days when closures were imposed) was correlated with changes in conflict mortality at the national level (correlation coefficient 0.52). Non-combatant conflict mortality and restrictions on movement were particularly high during the Second Intifada (2000–2004) (Fig. 1). Separate analysis also showed the measure worked well whether used weighted by regional population size or not.

We account for the lagged effect of conflict on subsequent health care utilisation. In our analysis of maternal health, the variable *intensity of conflict* refers to the average of the square root of the conflict mortality data 9 months before childbirth. We chose the average over the pregnancy to summarise the general trend over the period as no other cut off line would have been suitable for any of the outcomes variables. In the analysis of infant vaccination, *intensity of conflict* is the mean of the square root of conflict mortality rates during the first 8 months after delivery. The period was chosen because children should have received a third dose of OPV and DPT vaccines by the age of 6 months, according to the Palestinian Children Vaccination Programme schedule [37]. This construction of the variable allows accounting for effects other than temporary blockages, including the psychological impact that conflict intensity over a protracted period might have on an individual as well as the long-term consequences of supply shortages and infrastructure deficiencies.

*ANC* is a binary variable that records whether a woman attended at least five antenatal care visits during her pregnancy. Until 2016 the WHO guidelines recommended a minimum of 4 ANC visits for each pregnancy, revised to a minimum of 8 ANC visits in 2016 [38]. The distribution of ANC visits in oPt data suggests that the major drop in the distribution occurs in the value of 5 visits rather than 4, possibly reflecting the WHO guidelines in operation at the time. After conducting a sensitivity analysis of the various cut-off points, we chose the cut-off for this binary variable based on the actual distribution of the data rather than the contemporaneous guidelines as it approximated more closely the actual use.

*Caesarean delivery* indicates whether a woman gave birth by C-section or not. This indicator is used to define the quality of maternity care and is also an indicator of minimum availability of Emergency Obstetric Care [39]. In addition, this variable is increasingly being used as a measure of overmedicalisation of births when C-section is not performed for clinical need but instead due to supply or demand factors. For example, in the oPt there is a risk that elective C-sections may have been used for non-medical purposes to permit timed delivery to avoid the possibility of crossing checkpoints while in labour [7]. Within this context the interpretation of this variable needs to be cautious as both these instances could occur.

Finally, the variable *any pregnancy complications* is a binary variable equal to 1 if a woman reported experiencing any complications during her pregnancy and 0 otherwise. This included bleeding, hypertension and pre-eclampsia. This variable required harmonization as the list of pregnancy complications included in each survey varied. For example, hypertension and bleeding were consistently named using the same terminology across surveys, whereas we had to infer complications due to eclampsia by including categories within variables which mentioned symptoms related to eclampsia. Bleeding and hypertension were also analysed as single causes in separate models (not shown here). This variable represents the only health outcome that we could measure with the limited data across the surveys but it does give an important account of prenatal stress which has a negative impact on childbirth outcome's during conflict [14].

Vaccination schedules—for the DPT vaccine and the OPV—were reconstructed for the first 8 months of children’s lives. The variables *DPT and* OPV *vaccines* are binary variables that record whether a child received at least three doses of the given vaccine at or before their eighth month 6 months of age. We selected vaccination at eighth months because according to the immunization schedule for Palestine [40], a child is expected to receive the first three doses of vaccinations of DPT and OPV by the age of 6 months.

Table 4 presents summary statistics of the five response variables together with the share of missing values by region. For comparison, we have included the vaccination distributions at ages 9 and 12 months. Missing values for vaccination schedules could not be retrieved because older datasets used the same code for non-response and non-receipt of vaccine, making it impossible to distinguish whether a child was unvaccinated or the vaccination schedule was not reported by the mother. By age 12 months almost all children are vaccinated, with the exception of Central West Bank (CWB). Given the almost universal vaccination by age 12 months, issues of non-compliance with the vaccination schedule for oPt are likely to be due to vaccination delay rather than non-vaccination.

**Table 4:** Outcome variables, oPt by region 2000–14^a^

Prevalence by region	SGS %	NGS %	SWB %	CWB %	NWB %
DPT 6 months	74.1	67.8	51.1	56.5	60.1
DPT 9 months	97.6	96.9	95.2	93.6	96.4
DPT 12 months	98.9	97.9	97.5	95.6	97.6
OPV 6 months	73.5	67.5	48.6	49.5	57.7
OPV 9 months	97.2	97.1	93.4	86.1	95.1
OPV 12 months	98.6	97.9	96.7	91.1	96.5
Antenatal care (5+)	92.1	89.8	82.4	88.4	88.2
Caesarean delivery	9.8	12.9	10.3	13.1	14.2
Any complications^b^	44.1	41.3	52.5	52.6	61.3

^a^*SGS* South Gaza Strip, *NGS* North Gaza Strip, *SWB* South West Bank, *CBW* Central West Bank, *NWB* North West Bank

^b^Includes: bleeding, hypertension, pre-eclampsia, urinary infection

## Methods

The study used logistic regression for binary outcomes to explain the changing outcomes of four variables: child vaccination schedules; antenatal care; C-sections; and whether a woman experienced any complications during pregnancy. We analysed pooled data comprising 16,793 children with over 30,000 vaccination schedules, and the last pregnancy of 8477 women aged 15–45. We reconstructed data on C-sections and DPT vaccination with pooled data from the Demographic and Health Surveys for 2000–2014. Other explanatory variables included a wealth quintile—estimated using a Principal Component Analysis of material conditions (main water source, type of toilet, floor material, presence of iodised salt, household overcrowding) [41]. In addition, we included mother’s education, parity, child’s sex (only for vaccination), and mother’s age at delivery.

We accounted for unobserved heterogeneity for all the events occurring during the period which were not conflict-related, such as government change or health reforms, by using survey period as a fixed effects variable. For maternal health outcomes we considered the survey design for the modelling with sample weighting, whereas for the immunisation models we run a two-level model with random effects at household level. This is in order to account for multiple children within the household. Ideally we would have controlled for random effects at cluster level but not all surveys reported this information. The household level is the closest to a community effect possible with the available data. The model for a maternal health outcome is equal to:

1 $$ \Pr \left( MHO=1|X\right)\mathrm{Logit}\left( MHO=1|X\right)=\phi \left({X}^T\beta \right)\alpha +\left({X}^T\beta \right) $$

Where MHO is a Maternal Health Outcome (ANC, C-section, pregnancy complications) which is binary either 1 or 0; *ϕ* is the Cumulative Distribution Function, α is the intercept; *X*^*T*^ is the vector of regressors X and β are parameters estimated through maximum likelihood. The model for child immunization is equal to:

2 $$ \Pr \left({V}_{ij}=1|{X}_i\right)=\phi \left({X}_{ij}^T\beta +{z}_{ij}^T{b}_i\right) $$

Where V is the vaccination (DPT or OPT) and is binary; *i* is individuals; *j* households; z denotes a vector of covariates, possibly overlapping with X, having random effects b distributed normally. Other approaches, such as ARIMA models to account for the time series, were not been feasible since not have all the temporal (e.g.: monthly) information needed for both the outcomes and covariates was available.

### Limitations

This study has three key limitations, the most important of which is an inability to include more outcomes related to health status or healthcare access. For example, it would have been useful to account for child mortality and morbidity (eg: diarrhoea) but it was not possible to harmonise the data longitudinally due to missing information and inconsistent coding. Data limitations restricted the number and type of background characteristics that we could control for in our models; it would have been useful to include more information such as where people were living or their employment status.

Secondly, in our analyses we controlled for intensity of conflict but reforms and political changes which are not directly related to conflict both in Israel and the oPt could have had an impact on access to services, such as leadership changes in the Gaza Strip. However, we have controlled for survey period fixed effects to account for the unobserved heterogeneity of policy changes which we could not control for. This was the only feasible variable for this kind of pseudo longitudinal study and the strong correlation with other variables of conflict (e.g.: checkpoints closures) makes it an excellent candidate for the analysis. Lastly, our analyses did not include other indicators of conflict such as blockages and restrictions on movement. Again, this was due to data limitations. However, by averaging out the intensity of conflict throughout the period (i.e.: 9 months for women and 6 months for children) we do account for sudden changes in conflict dynamics. In addition, by measuring the lagged effect of conflict in the models, we capture the medium-term indirect impact of conflict. In this instance indirect impact includes delaying being vaccinated or getting ANC or as in the case of pregnant women, suffering from stress during the pregnancy and having an adverse outcome in the end. Finally, conflict intensity and the blockades (averaged as disaggregated data was not available) were highly correlated, reassuring us about the suitability of the way we had constructed the variable intensity of conflict.

## Results

**Fig. 2:**
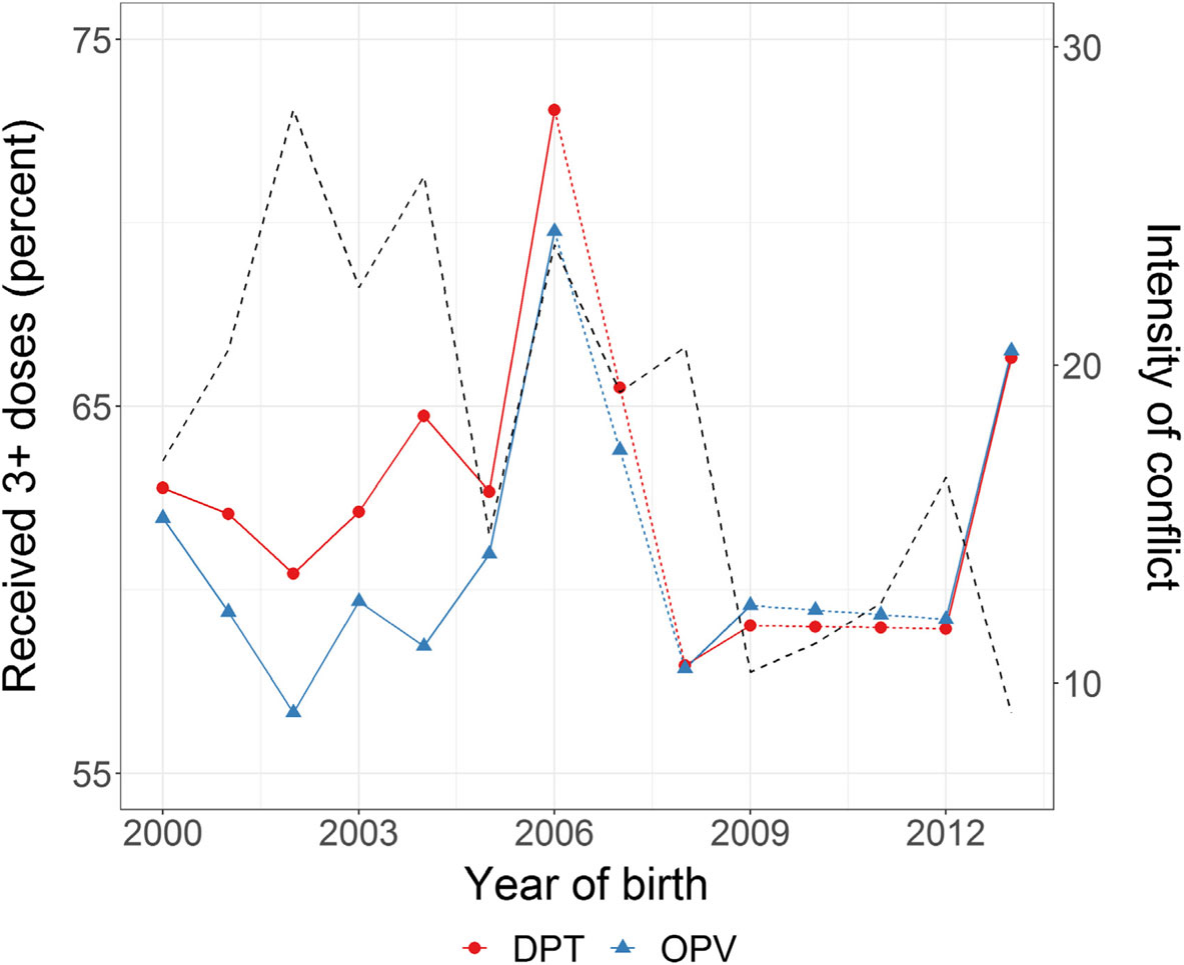
Intensity of conflict and immunisation by age 6 months oPt 200–14

**Fig. 3:**
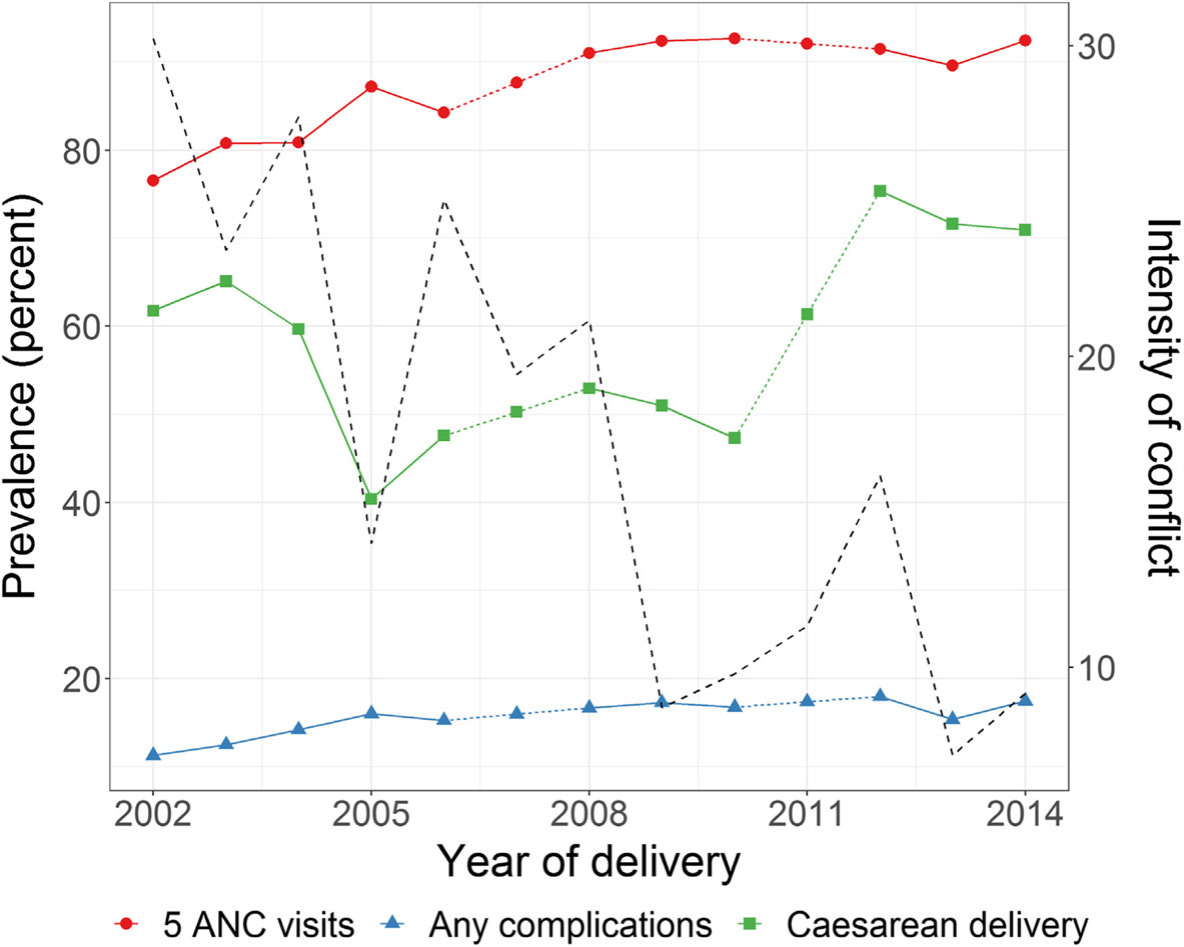
Maternal health care and intensity of conflict 2000–14

Vaccinations seem to have experienced several setbacks between 2000 and 2014 (Fig. 2), with a spike in 2006, followed by a considerable drop until 2013/14 when the numbers recovered. The data show a general increase in the prevalence of both C-sections and ANC since 2002, although with some stalling (Fig. 3). Complications during pregnancy show several setbacks during this period which seem to be in line with the reported conflict intensity trends.

When modelling maternal health outcomes in relation to intensity of conflict (Table 5) there is a strong negative correlation with ANC (− 0.102 *p* < 0.001) and a mild negative correlation with C-sections (− 0.045 *p* < 0.05). The negative correlation with complications during pregnancy is less clear. Complications decreased in 2006 and 2010 compared to 2004, however this is the survey data point most affected by the greatest intensity of conflict overall and this is evident in separate analysis we conducted by single surveys (not shown here). However, the overall trend does not seem to be affected and the negative impact is minor (− 0.062 *p* < 0.0001). We also ran separate models for bleeding and hypertension. While bleeding was not significant, hypertension showed a positive correlation with intensity of conflict (not shown here).

**Table 5:** Logistic models of women’s maternal health outcomes, oPt, 2000–2014

	ANC		Complications during pregnancy		Caesarean Delivery	
	B	SE	B	SE	B	SE
Intensity of conflict	−0.102***	0.026	−0.088***	0.018	−0.045**	0.021
Survey year						
2004	Reference					
2006	0.258***	0.065	−0.890***	0.056	−0.053	0.062
2010	0.604***	0.102	−0.894***	0.071	−0.036	0.082
2014	0.409***	0.133	0.157	0.098	0.082	0.109
Region^a^						
SWB	Reference					
CWS	0.233***	0.073	−0.094*	0.060	0.223***	0.067
NGS	0.457***	0.068	−0.280***	0.055	0.232***	0.064
NWB	0.313***	0.065	0.477***	0.049	0.393***	0.057
SGS	0.622***	0.072	−0.130	0.054	0.054	0.065
Maternal age at birth	− 0.003	0.005	−0.008	0.004	0.062***	0.004
Parity						
1	Reference					
2–3	−0.298***	0.070	−0.092	0.048	−0.276***	0.055
4–5	−0.246***	0.079	0.057	0.057	−0.565***	0.066
6+	−0.400***	0.103	0.167	0.079	−0.734***	0.090
Wealth quantile						
Poorest	0.052	0.076	0.115	0.066	0.121*	0.069
Poor	0.068	0.077	0.556***	0.056	0.089	0.063
Average	Reference					
Rich	−0.120*	0.071	0.078	0.052	0.024	0.060
Richest	−0.048	0.076	0.107	0.060	−0.037	0.067
Education						
Less than Secondary	0.014	0.049	0.031	0.038	0.077*	0.045
Secondary	Reference					
Some tertiary+	0.097	0.065	−0.132*	0.048	−0.032	0.055
*Constant*	0.849***	0.164	0.939***	0.126	−2.502***	0.143

^a^*SWB* South West Bank, *CWS* Central West Bank, *NGS* North Gaza Strip, *NWB* North West Bank, *SGS* South Gaza Strip

*** *p* < 0.001 ** *p* < 0.05 * *p* < 0.10

There were regional variations in the effect that intensity of conflict had on the outcomes with South West Bank reporting the strongest relationship between intensity of conflict and maternal outcomes such as ANC and C-section and the North West Bank to the highest levels of pregnancy complications (0.298 *p* < 0.001). In line with existing evidence, use of ANC declines with increasing parity [42]. Although parity is not significant for pregnancy complications, it shows a negative relationship with C-section. In addition, maternal age was only significant and positively associated with C-section (0.062 p < 0.001). Socio-economic characteristics such as education and wealth are not highly significant. The only noteworthy result is the increased risk of complications for the poorest quantile.

The patterns of the immunisation models differ only slightly in the overall intensity of the model coefficients across all the variables between the two models (Table 6). This is unsurprising since the immunisation schedule includes both DPV and OPT and the only reason for not reporting them at the same time could either be miss-reporting or shortage of supplies at the time of the vaccination. Intensity of conflict has a strong significant negative impact for both OPV (− 0.223 *p* < 0.001) and DPT (− 0.227 *p* < 0.001) vaccination. The period most affected is between the 2006 and 2010 surveys when the likelihood of being vaccinated declines.

**Table 6:** Multilevel logistic models children’s vaccinations, oPt 2000–2014

	OPV		DPT	
	B	SE	B	SE
Intensity of conflict	−0.223***	0.015	−0.227***	0.015
Survey year				
2004	Reference			
2006	−0.601***	0.051	−0.548***	0.051
2010	−0.502***	0.193	−0.559**	0.194
2014	0.196	0.196	0.128	0.197
Region				
SWB	Reference			
CWB	−0.047***	0.047	−0.161***	0.047
NGS	0.272***	0.052	0.253***	0.051
NWB	0.671***	0.050	0.692***	0.050
SGS	0.275***	0.053	0.271***	0.053
Maternal age at birth	−0.002	0.002	−0.001	0.002
Birth order				
1	Reference			
2–3	0.245***	0.036	0.144***	0.036
4–5	0.037*	0.037	0.041**	0.037
6+	−0.070	0.064	−0.178***	0.064
Wealth quantile				
Poorest	−0.096**	0.046	−0.098**	0.046
Poor	−0.062***	0.062	−0.148**	0.062
Average				
Rich	−0.061	0.048	−0.060	0.047
Richest	−0.124	0.050	−0.116	0.050
Education				
Less than secondary	−0.054	0.036	−0.058	0.036
Secondary				
Some tertiary+	0.278***	0.044	0.263***	0.044
Sex of child				
Female	Reference			
Male	0.102**	0.038	0.097**	0.037
*Constant*	1.420***	0.100	1.387***	0.100
*Intraclass correlation*	0.493***	0.083	0.495***	0.084

*** p < 0.001 ** p < 0.05 * p < 0.10

Socio economic characteristics show a lower propensity to use MCH services for poorer strata in both models and an insignificant difference for education. Regionally, the risk of vaccination is lower in central and Southern WB and male children are more likely to be vaccinated than females. Maternal age at birth is not significantly associated with the risk of vaccination whereas parity shows a higher likelihood of being vaccinated from parity 2 and above. For both vaccination models over 49% of the variance in the outcomes is explained by household variations in the outcome variable.

## Discussion

This study makes two novel contributions to our understanding of the impact of conflict on health. It presents the first longitudinal analyses of child and maternal health for the oPt. Second, it establishes a link between fluctuations in the intensity of conflict and maternal and child healthcare outcomes. The reconstructed data provide a unique source of information on trends in maternal and child healthcare utilisation in the region. Conflict intensity is key to understanding these changes over time. A more comprehensive measure of the intensity of conflict would also include restrictions on movement, but these data are not available at a regional level. A measure at national level would have not been representative of the impact of the conflict on health care access as the restrictions change dramatically across regions. This analysis would not be possible with any other conflict variable available for the oPt.

The results show the impact of conflict intensity on immunisation and access to maternal health care. Above all, the most important result is that preventative services such as ANC and immunisation were the most negatively affected by heightened conflict. Secondly locality is key in understanding the variations in effects both from a conflict intensity point of view mostly because of variations in access within the oPt due to blockages. Thirdly socio-economic circumstances do shield individuals from the impact of conflict. Finally, the negative result for pregnancy complications is an indication that during conflict, a lower utilization of services is reflected in self-reported data. We therefore need a more comprehensive set of indicators when looking at the impact of conflict on healthcare.

ANC and C-section both show a significant negative relationship with intensity of conflict. ANC during increased conflict might be considered a non-necessary medical visit adding stress at a time when blockages are increased and travel to healthcare might be more difficult and more dangerous [18]. The negative impact seems to have most affected Southern WB where the concentration of health facilities is the lowest and the area is more deprived compared to the rest of the WB.

By contrast, C-section is an indicator of several aspects of maternal health care, including emergency obstetric care, quality of care, and over-medicalisation [43, 44]. Potentially, an increase in C-sections could create risk for maternal and child health in situations where the system is precarious and care is sub-optimal, with the results we obtain from the models needing cautious interpretation. The increased prevalence of C-sections in a protracted conflict setting might result from the medicalisation of deliveries and efforts to control timing of births in a context of uncertainty and fear. However, the negative correlation with conflict is in line with the observed deterioration of the medical infrastructure, and/or health service competition to care for injuries, thus relegating childbirth to a secondary priority and undermining the availability of C-section for birthing women. Although availability of maternal health services has increased since 2000 an assessment of service provision in 2009 shows serious gaps in the continuum of maternal care [7]. Work force training, shortage of supplies and a progressive impoverishment of the health system all contribute to the deterioration of the overall health system and more specifically MCH services.

Another important result relates to locality. Within the WB, the South and Central WB are the regions most affected by conflict 2000–2014. In addition to the conflict, the Separation Wall and low density of higher level health centres mean that access is further negatively affected. The Gaza Strip is a very small area where distance to services is shorter than in the West Bank. While periodic attacks on the Gaza Strip and the ongoing siege have created dreadful living conditions, the Gaza Strip population is currently supported by the UNRWA which provides services for more than 2/3rd of the population, in addition to governmental and NGO services. However due to the recent (2018) US decision to cut UNRWA funding this has come into serious jeopardy [24]. Due to the relatively small distances and the absence of checkpoints within the GS (unlike the WB), and despite the siege, routine care such as ANC and immunization might occur at higher levels in the Gaza Strip compared to the WB. In contrast, more complicated procedures such as C-section requiring specialized care might not be available in the Gaza Strip, and require that patients are moved to the WB and East Jerusalem Hospitals [45]. This is in line with evidence that shows that access to more advanced procedures is more challenging for the Gaza Strip population both in terms of getting to the services as well as getting a permit to go to health services outside the Gaza Strip [22, 23, 45].

The only variable representing a health outcome, self-reported complications during pregnancy, shows a very small negative correlation with increased intensity of conflict. This might be due to wider issues with the data, such as underestimation due to lower use of services during more intense conflict which would lead to a lower detection of symptoms. This is in contrast with the overall results of the study where we find that access to health services is negatively affected by an increase in the intensity of conflict. Many symptoms during pregnancy are dismissed by women as simply being a fact of life due to being pregnant [46]. Such under-reporting can become even more pronounced in more deprived and conflict-afflicted areas, reflected in our results which show lower reporting of pregnancy complications for women in the Gaza Strip. Previous research on subjective health shows that Gaza Strip residents reported better subjective health than the West Bank but worse objective health, which is based on diagnosis rather than self-report [47].

Women are affected by conflict intensity irrespective of their socioeconomic status. Neither education nor household wealth show significant correlations with the health outcomes, in keeping with previous research [18]. Only in the models for DPT and OPV, the poorest report the lowest access showing that this group is affected regardless of the intensity of conflict. This lack of significance between socio-demographic characteristics and health outcomes in oPt is unusual compared to other settings and could suggest that the impact of conflict intensity might affect the supply of services as well as physical access. Under non-conflict conditions, access to planned C-section is usually higher among wealthier women, and emergency C-sections are higher among poorer and more disadvantaged women. However, the mechanisms that might drive the planned C-sections which have been on the rise in the oPt [48] do not seem to be driven by the wealthiest women as is the case in other countries [39]. Other literature also points to widespread distress among Palestinians during intensified conflict, as was the case also in other countries, where increases in C-sections during armed conflict were reported, suggesting that stress during pregnancy is implicated in the increases in C-section delivery rates [49, 50].

The impact of conflict on infant immunisation reflect findings from Iraq and Syria [16, 17], with increased conflict delaying or impeding access to immunisation. The closer association between vaccination and intensity of conflict as compared to maternal health outcomes might result from decisions to avoid potential risk (eg. checkpoint crossing) for non-urgent healthcare, or it could reflect reduced supplies and services during periods of increased conflict [5]. Our analyses show that the most acute effects for a range of health outcomes occurred when conflict intensity rose or dropped dramatically, suggesting that these periods are crucial for healthcare provision.

## Conclusions

Our approach was mainly focussed on demand indicators: three (ANC, DPT and OPV) are preventive interventions, one (c-section) is an intervention that can be both a result of birth complications but is also susceptible to demand by women and preferences of providers; and the fourth (pregnancy complication) is health status complications. The strongest effects of conflict were on uptake of preventive interventions, immunization and ANC. This result should be considered in future policy and in planning for possible heightened conflict periods.

Our analyses show a direct impact of increased conflict on access to MCH care. The literature highlights how major barriers to access include fears, frustrations, humiliations and possibly resignation of checkpoint crossings to seek healthcare [5]. When controlling for intensity of conflict, socio-economic characteristics do not seem to be a significant barrier to access to MCH as also shown previously [18]. Psychological barriers to access need to be better understood in order to design and implement community level interventions that can increase access to vital MCH services.

There is a shortage of specialised health care in the oPt [7], in particular paediatricians, obstetricians, and gynaecologists. Previous research has shown a low use of midwives and community health workers for maternal and child healthcare in the region. Midwives can provide a range of care, including ANC, routine delivery, postpartum care and vaccination, making services more accessible and at a lower cost to the health system. Global evidence on the benefits of midwife-led childbirth is well-established, not only in terms of care given but also in terms of cost-effectiveness and positive effect on equity [51]. Increased training and provision of midwifery care in oPt has the potential to increase access to and use of MCH services.

There is a need to re-assess how pregnancy, childbirth and infant care are provided in situations of protracted conflict [7, 14, 49]. Midwifery is certainly one option, as well as community health care providing basic services for both neonates and mothers. The tendency to rely on doctors and C-sections even if not needed in maternal and child healthcare might prove counterproductive in the oPt. There is a need to better understand how conflict impacts on childbirth outcomes and medicalisation of births both from the supply and demand side in terms of timing of childbirth but also the level of stress that can have on the whole process. In addition, telemedicine could be explored as a further mean to reach populations usually cut off either because of location or because of last minute check-points closures. This approach is at its infancy in Palestine [52] and MCH services could be challenging to be delivered in this way. However, further research is needed in particular to test how preventative services such as vaccinations, can be scaled up in order to avoid delays due to heightened conflict periods.

Community-based care can improve access to services in hard to reach populations, including populations that are hard to reach due to checkpoint or geographic barriers. For example, antenatal care visits and vaccinations can be provided by midwives or appropriately trained community health workers, assuming the supplies to maintain the vaccination chain. Macro-level influences, including the influence of the international community and funding are critical for the recruitment and training of more health workers and the availability of mobile service delivery in several areas of the oPt [26].

Finally, this paper approached the impact of conflict on healthcare from the demand side demonstrating how we can still conduct significant research with limited but yet rich data and obtaining more nuanced data on intensity of conflict. Future studies will need to examine the supply and demand sides together. This would entail a much more complex research design. Options would need to include key informant interviews, service logistics as well as budgetary data as well as research on restriction on movement doing quasi-archival data or using satellite imagery.

## Data Availability

Data are available on request from the Palestinian Central Statistical Bureau. We do not have permission to share.

## Appendix

**Table 7:** Non-combatant conflict mortality and Intensity of conflict variable

Year	South GS		North GS		South WB		Central WB		North WB	
	n	$\sqrt{n}$	n	$\sqrt{n}$	n	$\sqrt{n}$	n	$\sqrt{n}$	n	$\sqrt{n}$
2000	74	8.6	41	6.4	35	5.9	47	6.9	79	8.9
2001	111	10.5	68	8.2	73	8.5	47	6.9	154	12.4
2002	242	15.6	131	11.4	113	10.6	98	9.9	405	20.1
2003	167	12.9	203	14.2	37	6.1	18	4.2	148	12.2
2004	308	17.5	315	17.7	19	4.4	21	4.6	157	12.5
2005	45	6.7	58	7.6	7	2.6	6	2.4	67	8.2
2006	150	12.2	376	19.4	13	3.6	15	3.9	106	10.3
2007	135	11.6	160	12.6	8	2.8	19	4.4	57	7.5
2008	134	11.6	280	16.7	11	3.3	6	2.4	26	5.1
2009	18	4.2	24	4.9	7	2.6	6	2.4	4	2
2010	36	6	32	5.7	4	2	2	1.4	6	2.4
2011	45	6.7	59	7.7	2	1.4	4	2	3	1.7
2012	100	10	146	12.1	3	1.7	4	2	1	1
2013	5	2.2	4	2	8	2.8	10	3.2	9	3
2014	8	2.8	12	3.5	16	4	19	4.4	12	3.5

## Abbreviations

ANC: Number of antenatal visits
C-sections: Caesarean sections
CWB: Central West Bank
DPT: Diphtheria, pertussis, and tetanus
GIS: Geographical Information Systems
MCH: Maternal and child health care
NGOs: Non-Governmental Organisations
NGS: North Gaza Strip
NWB: North West Bank
oPt: Occupied Palestinian territory
OPV: Oral Polio Vaccine
SGS: South Gaza Strip
SWB: South West Bank
UNRWA: UN Relief and Works Agency for Palestine

## Footnotes

1 Defined as defined as the number of deaths following an armed conflict minus the number of deaths that would have occurred in the same period if the war had not occurred.

2 For more information on the history of the Palestinian territories and its impact on the health systems see Giacaman et al., 2009

3 For a more up to date timeline of maps of blockages and closures please refer to https://www.ochaopt.org/ and http://www.btselem.org/

4 https://www.map.org.uk/downloads/no-more-impunity%2D%2Dgazas-health-sector-under-attack.pdf; http://www.alhaq.org/advocacy/topics/gaza/855-briefing-note-vii-illegal-attacks-on-health-facilities-and-hospitals
